# Finding an easy way to harmonize: a review of advances in clinical research and combination strategies of EZH2 inhibitors

**DOI:** 10.1186/s13148-021-01045-1

**Published:** 2021-03-24

**Authors:** Chen Li, Yan Wang, Yueqing Gong, Tengrui Zhang, Jiaqi Huang, Zhen Tan, Lixiang Xue

**Affiliations:** 1 Department of Radiation Oncology, Peking University Third Hospital, Beijing, 100191 China; 2 Center of Basic Medical Research, Institute of Medical Innovation and Research, Peking University Third Hospital, 49 North Garden Road, Haidian District, Beijing, 100191 China; 3 Department of Bone and Joint Surgery, Peking University Shenzhen Hospital, Shenzhen, 518036 People’s Republic of China

**Keywords:** EZH2, EZH2 inhibitors, Combination therapy, Immunotherapy, Chemotherapy, Targeted therapy, Endocrine therapy, Cancer epigenetics

## Abstract

Enhancer of zeste homolog 2 inhibitors (EZH2i) have garnered increased attention owing to their anticancer activity by targeting EZH2, a well-known cancer-promoting factor. However, some lymphomas are resistant to EZH2i, and EZH2i treatment alone is ineffective in case of EZH2-overexpressing solid tumors. The anti-cancer efficacy of EZH2i may be improved through safe and effective combinations of these drugs with other treatment modalities. Preclinical evidence indicates that combining EZH2i with other therapies, such as immunotherapy, chemotherapy, targeted therapy, and endocrine therapy, has complementary or synergistic antitumor effects. Therefore, elucidating the underlying mechanisms of the individual constituents of the combination therapies is fundamental for their clinical application. In this review, we have summarized notable clinical trials and preclinical studies using EZH2i, their progress, and combinations of EZH2i with different therapeutic modalities, aiming to provide new insights for tumor treatment.

## Background

Enhancer of zeste homolog 2 (EZH2) is a classical epigenetic regulator of tumor genesis, metastasis, and prognosis. In recent decades, a series of EZH2 inhibitors (EZH2i) have been discovered, reported, and tested in clinical trials; these include tazemetostat, GSK126, CPI-1205, PF-06821497, and SHR2554. In early 2020, tazemetostat was approved by the US Food and Drug Administration (FDA) for use in treatment of adults and adolescents with locally advanced or metastatic epithelioid sarcoma. However, clinical trials targeting EZH2 in other solid tumors did not show satisfactory results. For example, at a maximum-tolerated dose (MTD; 2800 mg twice weekly), defined by a dose escalation study, GSK126 showed only moderate antitumor activity [1].

In recent years, combination therapy has become the hallmark of cancer treatment considering the significantly decreased incidence of tumor recurrence and disease progression compared to monotherapies. Therefore, in current review, we summarizes the results of clinical trials using EZH2i and the effects of EZH2i combined with other therapeutic modalities in cancer treatment to enhance treatment efficacy compared to monotherapies. Moreover, this review provides a deeper insight into the clinical potential of the combination therapies against tumors.

## Overexpression and mutation of EZH2 is correlated with tumor progression

EZH2 is the catalytic subunit of polycomb repressive complex 2 (PRC2), which catalyzes histone H3K27me3 through its C-terminal SET domain to promote chromatin compression and silence its target genes [2, 3]. EZH2 expression is reportedly increased in murine prostate cancer (PCa) [4] and lymphoma cell lines [5] as well as in human primary tumor cells, such as breast cancer [6], melanoma [7], and pancreatic cancer [8]. Due to its high expression in several cancer cells and the downstream of pPB-E2F pathway, EZH2 is universally recognized as a marker of proliferation and a bona fide oncogene [9].

In addition to overexpression, EZH2 mutation was also confirmed to be highly correlated with tumor progression. A single tyrosine 641 (Tyr641) in the SET domain of EZH2 is replaced in 7.2% follicular lymphoma (FL) and 21.7% germinal center B-cell-like (GCB) subtype diffuse large B-cell lymphoma (DLBCL) [10]. Tyr641 is a functional acquired EZH2 mutation that promotes the growth of B-cell lymphoma and melanoma, characterized by high expression levels of the anti-apoptotic protein Bcl2 and loss of the tumor inhibitor p53 [11]. In addition, the mutation of EZH2 alanine 677 to glycine (A677G) in B-cell lymphoma cell lines and primary tumor specimens resulted in aberrantly elevated H3K27me3 [12]. Hypertrimethylation can also be driven by the mutation of EZH2 alanine 687 to valine (A687V) in Non-Hodgkin’s lymphoma (NHL) [13]. Other mutation hot spots of EZH2, including A682 and A692, were identified in FL subsequently [14]. These studies have shown that EZH2 mutations may drive the proliferation of cancer cells, making EZH2 a molecular therapeutic target in patients harboring these site mutations.

## Competitive inhibition of EZH2 methyltransferase activity

**Fig. 1:**
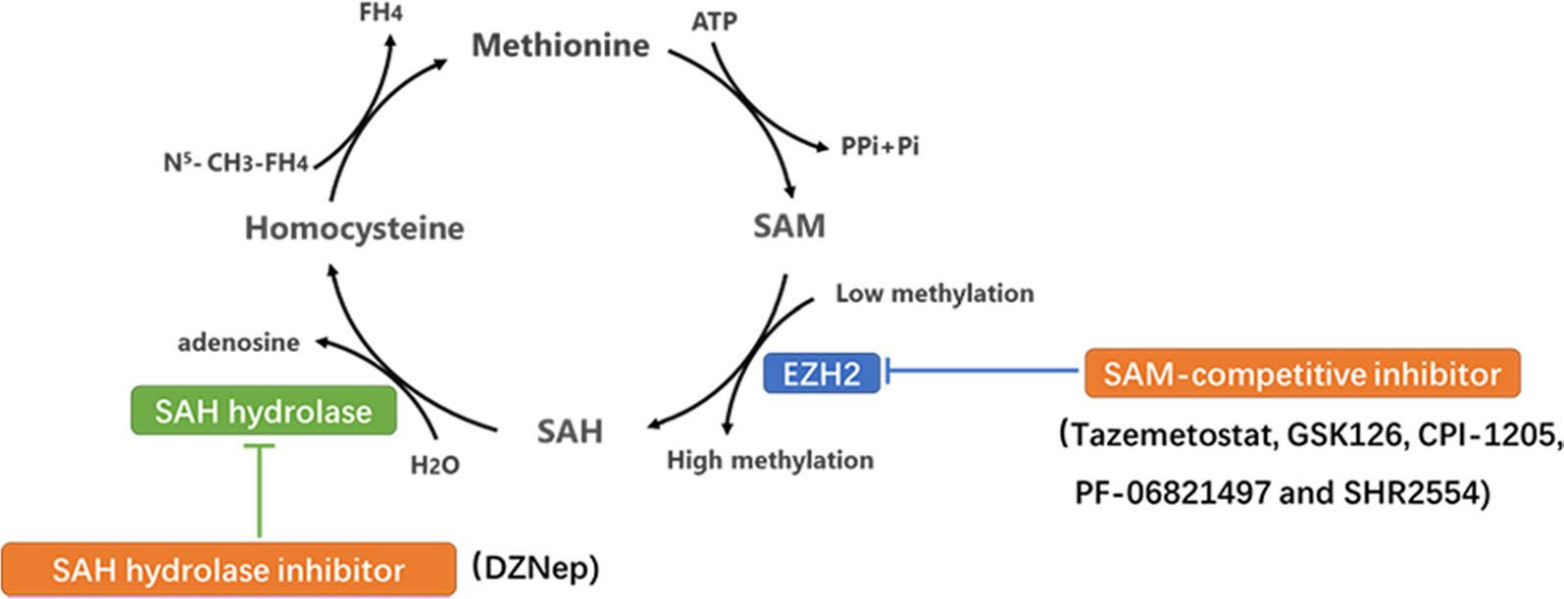
Two types EZH2i through suppressed key steps of Methionine cycle. (1) SAH hydrolase inhibitor inhibits SAH degradation into adenosine and homocysteine to cause methyl accumulation, which suppresses methionine cycling production of methyl and inhibits EZH2 enzyme activity indirectly. (2) SAM-competitive inhibitor partially occupies the site for the co‐substrate SAM in the binding pocket of EZH2 to deprive EZH2 the use of methyl, such as tazemetostat, GSK126, CPI-1205, PF-06821497 and SHR2554. These inhibitors have more direct characteristic compared to the SAH hydrolase.

Overexpression or functional acquired mutations of EZH2 promote cancer progression by enhancing the function of methyltransferase [15]. EZH2 modifies its target genes with the methyl group provided by S-adenosylmethionine (SAM) in the methionine cycle. SAM is demethylated to form S-adenosylhomocysteine (SAH), which is decomposed into homocysteine and adenosine, and the follow-up cycle provides a continuous supply of methyl groups to the biochemical reaction. Blocking the production and utilization of methyl groups is a key step in inhibiting the enzyme activity of EZH2. Therefore, various EZH2i has been developed to target the methionine cycle; these include competitive inhibitors with SAM binding to the EZH2 enzyme pocket, decreasing its utilization and suppressing SAH degradation to inhibit SAM production (Fig. 1).

### SAH hydrolase inhibitor

DZNep, the first identified EZH2i, inhibits SAH hydrolase. It inhibits the hydrolysis of SAH and causes its accumulation, thus blocking the release of the methyl group from SAM, thereby exerting antitumor effects [16, 17].

### SAM-competitive inhibitor

SAM-competitive EZH2i are a series of novel, highly potent small molecules that inhibit EZH2 enzyme activity. These molecules have a 2‐pyridone moiety, which is crucial for enzyme inhibition and partially occupies the site for the co‐substrate SAM in the binding pocket of the enzyme. Different drugs have different linking support substructures of 2‐pyridone warheads, such as a bicyclic heteroaromatic ring, including indazole [18] (e.g., EPZ005687 and UNC1999) and indole [19] (e.g., GSK126, EI1, and CPI‐1205), or a simple monocyclic (hetero) aromatic ring, including tazemetostat and MC3629. Different substitutions at the support moiety influence the pharmacokinetics and pharmacodynamics of the compounds as well as their water solubility [20].

## EZH2i as promising epigenetic drugs

Currently, five EZH2i, including tazemetostat (Tazverik, US); GSK126 (GlaxoSmithKline, UK); CPI-1205 (Constellation, US); SHR2554 (HengRui Medicine Co., LTD, China); and PF-06821497 (Pfizer, US), have entered into clinical trials (Table 1). Among these, tazemetostat was approved by the FDA in January 2020, making it the first EZH2i drug for the treatment of adults and adolescents aged ≥ 16 years with advanced or metastatic epithelioid sarcomas, which are not suitable for surgery and other standard treatments [21, 22].

**Table 1:** Current EZH2 inhibitors in clinical

Drug	Condition	Phase	Combined Drug
Tazemetostat (Epizyme, US)	Maligant Rhabdoid Tumors, Solid Tumor, Epitheliod Sarcoma, Bladder Cancer, Prostate Cancer, Lymphoma, Urothelial Carcinoma	Approved	Abiraterone/prednisone, Placebo Omeprazole, Repaglinide Pembrolizumab, Enzalutamide Fluconazole, Doxorubicin
GSK2816126 (Glaxosmithkline, UK)	Myeloma Solid Tumors Non-Hodgkin’s Lymphoma	I	–
CPI-1205 (Constellation, US)	B-Cell Lymphom Prostate Cancer Advanced Solid Tumors	I/II	Ipilimumab Enzalutamide Abiraterone/prednisone
SHR2554 (HengRui, China)	Lymphoma Breast cancer Prostate Cancer	I/II	SHR3680 SHR1701 SHR3680
PF-06821497 (Pfizer, US)	Small cell lung cancer Follicular Lymphoma, DLBCL Castration Resistant Prostate Cancer	I	–

### Tazemetostat

Tazemetostat, the first EZH2i approved by the FDA, is a potent, selective, and orally bioavailable small-molecule inhibitor of EZH2 enzymatic activity [23]. It can effectively inhibit the enzymatic activity of both wild-type EZH2 and EZH2 with catalytic domain mutation. In vitro, tazemetostat inhibits human lymphoma cell lines in a dose- and time-dependent manner. In vivo, tazemetostat inhibits tumor growth in xenograft-bearing mice with EZH2-mutant NHL, resulting in complete and sustained tumor regressions with lower H3K27me3 levels. Mice were dosed orally 2 or 3 times daily with tazemetostat for 28 days until the tumor volumes were about 120 mm^3^. Even 63 days after completion of EZH2i treatment, previously tumor-bearing mice remained tumor free in the two EZH2-mutant xenograft models. These data portend the utility of tazemetostat as a potential treatment for these genetically defined cancers [24]. Moreover, a phase II study of tazemetostat in patients with relapsed or refractory B-cell NHL harboring EZH2 mutations is ongoing (NCT03456726).

Until now, two results of tazemetostat clinical trials have been reported:

1. One is a first-in-human, open-label, phase I study of EZH2i in patients with relapsed or refractory B-cell NHL and advanced solid tumors. The report indicated that tazemetostat was effective against B-cell NHL and solid tumors. 8 of 21 patients with B-cell NHL and 2 of 43 patients with solid tumors had durable objective responses, including complete responses, supported by negative ^18^F-FDG-PET [25].
2. Another positive result for tazemetostat in patients with EZH2 mutation relapsed or refractory FL was reported at the 23rd European Hematology Association Congress. The study results indicated that patients who carried EZH2 mutations were more sensitive to tazemetostat compared to those with wild-type EZH2. Of 28 patients, 3 patients with EZH2 mutations responded completely, while 17 responded partially. This study also supported the notion that the EZH2 mutation status may be a biomarker to identify patients with FL who might benefit from tazemetostat treatment [26].

In these two studies, responses in solid tumors among patients were assessed by the investigators according to the Response Evaluation Criteria in Solid Tumors version 1.128 and the 2007 International Working Group criteria for patients with malignant lymphoma [27].

### GSK126

GSK126, a potent, highly selective, SAM-competitive, small-molecule inhibitor of EZH2 methyltransferase activity, decreases H3K27me3 levels globally and reactivates silenced PRC2 target genes; however, a change in dose does not influence the EZH2 protein level [28]. In 2012, GSK126 was identified as an effective compound that inhibited the proliferation of EZH2 mutant DLBCL cell lines. Moreover, in mice bearing Karpas-422 xenograft tumors, marked tumor regression was noted after treatment with GSK126 [29].

In 2014, the first phase I study of GSK126 clinical trial was carried out. Patients with relapsed/refractory DLBCL, transformed FL, other NHLs, solid tumors, and multiple myeloma (MM) were recruited in a clinical trial (NCT02082977). However, the outcome of the GSK126 clinical trial was unsatisfactory, as only one patient with GCB-DLBCL partially responded to treatment, while more than half of the patients had progressive disease without any changes of the baseline H3K27me3 in the peripheral blood mononuclear cells. Only a modest antitumor activity was detected at the tolerable doses of GSK126; thus, further clinical trials for this drug are not recommended [1].

### CPI-1205

CPI-1205 is a highly potent EZH2i (biochemical IC50 = 0.002 μM, cellular EC50 = 0.032 μM) showing higher selectivity for EZH2 than EZH1. CPI-1205 demonstrated robust antitumor effects in a Karpas-422 xenograft model at a dose of 160 mg/kg twice daily, showing improved cellular activity and good oral bioavailability [30]. Thus, it has been recommended for phase I clinical trials on B-cell lymphomas (NCT02395601).

### PF-06821497

In 2018, PF-06821497 was first identified as an EZH2i with adequate EZH2 inhibitory potency as well as physical and chemical properties such as thermodynamic solubility. In addition, it also showed robust tumor growth inhibition activity in a mouse Karpas-422 xenograft model when administered at a dose of 100 mg/kg twice daily and a strong association with pharmacodynamic effects, such as decreased H3K27me3 level, in tumors [31]. Currently, PF-06821497 is under phase I clinical trials for treatment of relapsed/refractory small cell lung cancer, castration resistant prostate cancer (CRPC), and FL (NCT03460977).

### SHR2554

SHR2554 is an orally administered small-molecule inhibitor of EZH2 developed by the Chinese pharmaceutical company Hengrui. Although there is no record of preclinical experiment, the safety of a single oral dose of SHR2554 in healthy subjects has already been evaluated (NCT04335266). SHR2554 has been investigated as a single-agent therapy in relapsed or refractory mature lymphoid neoplasms (NCT03603951). Moreover, it was also combined with the androgen receptor (AR) antagonist SHR3680 in patients with metastatic CRPC (mCRPC) (NCT03741712) and combined with SHR1701, inhibiting both programmed cell death ligand-1 (PD-L1) and transforming growth factor-β (TGF-β) II in advanced solid tumors and B-cell lymphomas (NCT04407741).

## Combined clinical EZH2i and other cancer treatment modalities

Similar to other tumor treatment modalities, EZH2i has their limitations. EZH2i activates insulin-like growth factor 1 receptor (IGF-1R), MEK and phosphoinositide-3-kinase (PI3K) pathways, resulting in lymphomas with acquired resistance to EZH2i [32]. In addition, clinical trials have shown that the antitumor activity of EZH2i alone is ineffective in EZH2 overexpressing solid tumors, such as prostate cancer, head and neck cancer, ovarian cancer, and soft tissue sarcoma [1].

Recently, accumulating evidence demonstrated that combinations of EZH2i with other therapies, including immunotherapy, chemotherapy, targeted therapy, and endocrine therapy, showed more robust antitumor effects that were complementary or synergistic (Table 2).

**Table 2:** The different therapeutic combinations with EZH2 inhibitor and the EZH2 targeted genes involved combination therapy

Cancer therapy	Therapeutic method	EZH2 target genes	Cancer types	Refs
Immunotherapy	Anti-CTLA-4	–	Bladder cancer	[38]
	Anti-PD-L1	CD274, IRF1	Hepatocellular carcinoma	[40]
	Anti-PD-1	NLRC5, CIITA	Head and neck cancer	[41]
	Anti-MDSCs	–	Colon cancer	[48]
Chemotherapy	Docetaxel	Nanog, Sox2, CD44	Prostate cancer	[55]
	Etoposide	BRG1, EGFR	Lung cancer	[56, 57]
	Cisplatin	–	Cervical cancer	[58]
		MiR-137	Ovarian cancer	[59]
		–	Osteosarcoma	[60]
	Temozolomide	MDR, MRP, BCRP	Glioblastoma	[61]
	Doxorubicin, Melphalan	ATM	Multiple myeloma	[64]
	Epirubicin, Mitomycin C	P53, ATM, FOXO3	Hepatocellular carcinoma	[65]
Targeted therapy	PARP inhibitor	MAD2L2	Ovarian cancer	[74]
		HOXA9, DAB2IP	Breast cancer	[75]
	HDAC inhibitor	P16, P21, P27, FBXO32	Acute myeloid leukemia	[78]
		–	Lymphomas	[80]
		NKD1, PPP2R2B	Lung cancer	[81]
		P21	Ovarian cancer	[82]
Endocrine therapy	Anti-androgen	PSA, TMPRSS2	Prostate cancer	[85]
		AR	Prostate cancer	[86, 87]
	Anti-estrogen	GREB1	Breast cancer	[90]

### EZH2i combined with immunotherapy

Epigenetic modifications can influence the reactivity of immune therapy through DNA or histone modifications [33]. For instance, DNA methyltransferase 1 (DNMT1)-mediated DNA methylation at the promoter regions of the genes encoding the tumor Th1-type chemokines CXCL9 and CXCL10 represses their transcription and translation in ID8 ovarian cancer in C57/BL6 mice. Consequently, cytotoxic T cell trafficking into the tumor microenvironment decreases. Similarly, through epigenetic reprogramming, EZH2 regulates differentiation and plasticity of T cells to shape the immune landscape in tumor microenvironments [34]. Based on this study, combing EZH2i with immunotherapy could provide a more effective cancer treatment.

#### EZH2i improves the efficiency of immune therapies

**Fig. 2:**
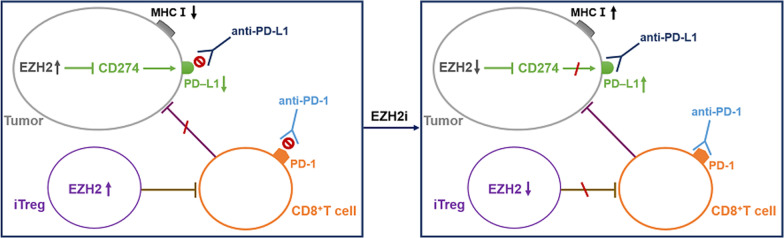
Inhibition of EZH2 improved the effect of blocking immune checkpoint. (1) In tumor cell, high level EZH2 can inhibit CD274, the transcription factor of PD-L1. Tumor cell with low expression of PD-L1 showed resistance to anti-PD-L1 therapy, which can recover the sensitivity after inhibition EZH2. (2) In iTregs, EZH2 overexpression enhances its suppression to CD8^+^T cells that cannot kill tumor cells. Addition of EZH2 inhibitors can restore CD8^+^T cells activity to enhance antitumor ability. 3) EZH2 overexpression in tumor cells inhibits the expression of MHC I, and influences the efficacy of anti-PD-1 treatment. Treatment with EZH2 inhibitor improves the efficacy of anti-PD-1 therapy.

Immune checkpoint blockade (ICB) therapies, such as treatments with anti-programmed cell death-1 (anti-PD-1), anti-programmed cell death-ligand 1 (anti-PD-L1), and anti-cytotoxic T lymphocyte antigen 4 (anti-CTLA-4), have achieved remarkable success in treating multiple types of cancer [35]. However, due to the heterogeneity of tumors and the differences in individual immune systems, primary and acquired resistance to ICB therapies limit their efficacy [36]. Epigenetic alterations may be a major factor underlying the heterogeneity of tumors and the differences in individual immune systems. Thus, an effective combination of immune checkpoint inhibitors and therapeutic agents targeting epigenetic changes may have potential to retrain the immune system and improve the clinical outcomes of ICB therapy [37] (Fig. 2).

Ipilimumab, an anti-CTLA-4 antibody, increases EZH2 expression in peripheral CD4^+^ T cells in patients; thus, inhibiting EZH2 expression in T cells may increase the effectiveness of anti-CTLA-4 therapy. As expected, a mouse model with genetic depletion of *EZH2* in Tregs (FoxP3^cre^EZH2^fl/fl^ mice) exhibited robust anti-tumor ability and more infiltration of effective CD8^+^ T cells secreting high levels of interferon (IFN)-γ, granzyme B, and tumor necrosis factor-α (TNF-α). Pharmacological inhibition of EZH2 using CPI-1205 could also enhance the activity of effector T cells by diminishing the suppressive activity of Tregs. Moreover, treatment of tumor bearing mice with CPI-1209 and anti-CTLA-4 resulted in a smaller tumor volume and longer survival duration than in those in the control groups [38, 39].

Anti-PD-L1 is another immune checkpoint inhibitor, which is based on the expression of PD-L1 on tumor cells. Recently, in vitro experiments revealed that EZH2 negatively regulated PD-L1 expression on hepatoma cells by upregulating the H3K27me3 levels in the promoter regions of *CD274*, which encodes PD-L1, and IFN regulatory factor 1 (IRF1). Inhibition of EZH2 can increase PD-L1 expression on the surfaces of tumor cells to improve the efficiency of anti-PD-L1 therapy [40]. Thus, the combination of EZH2i and PD-L1 blocker showed greater effectiveness in treating liver cancer.

Although anti-PD-1 therapeutics improve survival in patients with recurrent head and neck squamous cell carcinoma (HNSCC), patients with other types of cancer do not benefit from this treatment due to low response rates. Researchers have found that the addition of EZH2i to the therapeutic regimen can improve the efficacy of anti-PD-1 treatment. In an anti-PD-1-resistant model of HNSCC, tumor growth was suppressed through treatment with a combination of EZH2i and anti-PD-1. Inhibition of EZH2 significantly up-regulated MHC I expression by enhancing antigen presentation both in vitro in HNSCC lines and in vivo in a mouse model. In addition, EZH2 inhibition increased antigen-specific CD8^+^ T cell proliferation, IFN-γ production, and tumor cell cytotoxicity [41]. Moreover, another study showed that EZH2 inhibition relieved the suppression of NLRC5 and CIITA expression mechanistically, leading to the restoration of MHC expression and T cell infiltrates in DLBCL [42]. Thus, combination therapy using EZH2i and anti-PD-1 may improve anti-tumor treatment efficacy.

Besides ICB, adoptive cell transfer therapies have become new strategies to initiate and improve the treatment efficacy for anti-hematological cancers, such as acute lymphoblastic leukemia [43]. However, clinical treatments of solid tumors with these approaches have been less impressive due to difficulty in selecting a suitable target antigen and lower T cell infiltration [44]. To overcome this problem, a study evaluated combination therapy with EZH2i and adoptive T cell transfer and found that the tumor growth suppression was better after combination therapy than after either approach alone in ovarian tumor-bearing mice. Moreover, EZH2 expression in tumors is negatively associated with the population of tumor-infiltrating CD8^+^ T cells and patient outcome, thus providing a rationale for the combination of EZH2i and adoptive T cell transfer therapy [45].

#### Immune therapy compensates for the disadvantages of EZH2i

In multiple NHL cell lines, B cell maturation and activation are highly related to tumor progression. One study found that inhibition of EZH2 in both mutant and wild-type NHL leads to increased B-cell maturation and a greater dependence on B-cell activation signaling, as evidenced by the increased expression of B-cell maturation regulators such as PRDM1/BLIMP1 [46]. Interestingly, combining tazemetostat with B cell activation inhibitors (e.g., BTKi, SYKi, GRAGs, ibrutinib) showed synergistic benefits in both EZH2 mutant and wild-type DLBCL [47].

Moreover, EZH2i can re-shape the tumor immune environment, where effector T cells are suppressed but suppressor immune cells are activated. One study found that the anti-tumor ability of GSK126 showed different tendencies in wild-type mice and an immune-deficient mouse model bearing LCC and MC38 cell lines. Analysis of the change in immune cells infiltrating the tumor microenvironment revealed increased numbers of myeloid-derived suppressor cells (MDSCs) and decreased numbers of CD4^+^ T cells and IFN^+^ CD8^+^ T cells in WT-mice. Therefore, inhibition of MDSCs infiltrating the tumor immune environment can recover the activity of CD8^+^ T cells. A previous study reported that gemcitabine and 5-fluorouracil inhibit MDSCs. Therefore, the combination of GSK126 and MDSC-suppressors, such as anti-Gr1 antibody and gemcitabine/5-fluorouracil, can improve the anti-tumor ability of the treatment and overcome the deficiency of EZH2i [48].

### EZH2i combined with chemotherapy

Chemotherapy is considered the gold standard for the treatment of most cancers, including cervical cancer [49], breast cancer [50, 51], and prostate cancer [52]. However, chemo-resistance results in poor treatment efficacy and needs to be overcome.

Histone modification and chromatin remodeling are important events in response to DNA damage. However, cancer cells acquire resistance through epigenetic events ensuing DNA damage and repair, thus facilitating the evasion of cancer cells from programmed cell death [53, 54]. Therefore, a combination of targeted therapy and chemotherapy is likely an effective approach to overcome monotherapy resistance.

#### EZH2i improves cytotoxicity of chemotherapy

Overexpression of EZH2 has been confirmed in different cancer types, such as prostate cancer, and is involved in drug resistance [55]. Targeting EZH2 could overcome chemotherapeutic drug resistance.

After a certain period of docetaxel (Doc) treatment, both LNCaP and CWR22Rv1 prostate cell lines exhibited Doc resistance accompanied by upregulated EZH2 expression. Interestingly, EZH2 inhibition by DNZep or its miRNA mimics can re-sensitize resistant cell lines to Doc treatment [55]. Thus, targeting EZH2 may improve the efficacy of Doc treatment.

In addition, high EZH2 expression correlates with a poor prognosis in non-small cell lung cancers (NSCLC). Inhibiting EZH2 enzyme activity makes two NSCLC cell lines more sensitive to etoposide, which is a common chemotherapeutic drug. Moreover, if the lung cancer cells harbor inactivating mutations in BRG1 (SMARCA4) or activating mutations in epidermal growth factor receptor (EGFR), combination treatment with etoposide and EZH2i shows better efficacy with respect to impeding tumor growth both in vitro and in xenograft models than in cancer cells with wild-type EGFR and BRG1 [56, 57].

Another study showed that inhibiting endogenous EZH2 expression effectively reversed cisplatin resistance and increased cisplatin sensitivity in cisplatin-resistant cervical cancer cells by upregulating Dicer expression [58]. In ovarian cancer, c-Myc enhances the expression of EZH2 by directly suppressing miR-137, which targets EZH2 mRNA in resistant cells. Thus, increased expression of EZH2 activates cellular survival pathways, resulting in cisplatin resistance. Inhibition of the c-Myc-miR-137-EZH2 pathway leads to re-sensitization of resistant cervical cells to cisplatin [59]. Similar to cervical cancer, inhibition of EZH2 enhances cisplatin-induced apoptosis in osteosarcoma cells by improving the cisplatin-mediated cytotoxic effect [60].

In temozolomide (TMZ)-resistant U251 and U87 clones, EZH2 is highly expressed, suggesting that it plays an important role in multi-drug resistance and that EZH2i can enhance the effect of TMZ in multi-drug-resistant glioblastoma [61]. In patients with pancreatic cancer, the combination of DZNep and gemcitabine enhances the anti-proliferation effect of gemcitabine compared to gemcitabine monotherapy [62].

#### EZH2i increases chemotherapy efficacy by influencing DNA damage

Eliciting DNA damage and preventing DNA repair are the main effects of chemotherapeutic drugs, including doxorubicin and melphalan [63]. Consequently, enhancing DNA damage in tumor cells could improve the anti-tumor effect. Recently, in vitro experiments confirmed that targeting EZH2 with minimal toxic concentrations of GSK126 markedly aggravated the DNA damage response associated with apoptosis in MM cells, thus forming the rationale for the combination regimen of EZH2i with DNA-damaging agents in the treatment of MM [64].

Epirubicin and mitomycin C are used in the treatment of hepatocellular carcinoma (HCC). Our previous study investigated the biological and mechanistic roles of EZH2 in the DNA damage response induced by epirubicin and mitomycin C in HCC, revealing that EZH2 targeted genes involved in DNA damage, such as *p53*, Ataxia telangiectasia mutated (*ATM*), and Forkhead box O3 (*FOXO3*). Inhibition of EZH2 by shRNA or its inhibitor GSK126 significantly promoted chemotherapeutic drug-induced genotoxicity and increased HepG2 cell chemosensitivity. Therefore, targeting EZH2-induced p53 expression was associated with increased drug sensitivity in HCC cells [65]. These findings suggest that the link between epigenetic modifications by EZH2 and DNA damage induced by chemotherapeutic drugs may represent a new strategy for increasing the sensitivity of chemotherapeutic agents.

Overall, the combination of EZH2i and chemotherapies have synergistic efficacy, which provides a guideline for the use of EZH2i and chemotherapeutic drugs more rationally and effectively.

### EZH2i combined with other targeted therapies

Targeted therapy refers to revolutionized therapeutics that interfere with specific molecules to block cancer growth, progression, and metastasis [66]. Many of the FDA approved molecular targeted therapies have shown clinical success in the treatment of myriad cancer types, including breast cancer [67], leukemia [68], colorectal cancer [69], lung cancer [70], and ovarian cancer [71]. At the same time, a combination of two targeted treatments has become a trend in current clinical therapy.

#### EZH2i combined with poly ADP-ribose polymerase (PARP) inhibitor

PARP inhibitors (PARPi) have been used to treat patients with BRCA-mutant cancers lacking homology-directed DNA repair; however, drug resistance remains a clinical hurdle [72]. Research has revealed the epigenetic mechanism of PARPi resistance, and combining PARPi with EZH2i can improve the therapeutic effect [73].

For example, a recent study reported that the inhibition of EZH2 can sensitize ovarian cancer cells with CARM1-high expression, a homologous recombination (HR) proficient ovarian cancer, to PARPi in both orthotopic and patient-derived xenografts. Moreover, CARM1 promotes MAD2L2 silencing by driving the switch from the SWI/SNF complex to EZH2 through methylation of the BAF155 subunit in the SWI/SNF complex on the MAD2L2 promoter. Therefore, EZH2 inhibition can up-regulate MAD2L2 expression to decrease DNA end resection, ultimately causing mitotic catastrophe in PARPi-treated HR-proficient cells [74].

Another rationale for combining PARPi and EZH2i is that PARP1 interacts with and attaches poly-ADP-ribose (PAR) chains to EZH2 mechanistically. Protein poly ADP-ribosylation (PARylation) of EZH2 by PARP1 induces PRC2 complex dissociation and downregulation of EZH2 expression and H3K27me3. In contrast, alkylating DNA damage-induced EZH2 downregulation is attenuated in PARPi-treated cells, thereby promoting EZH2-mediated gene silencing unlike in PARPi-untreated cells. Moreover, the addition of EZH2i further sensitizes BRCA-mutant breast cells to PARPi [75].

#### EZH2i combined with histone deacetylase (HDAC) inhibitors

Histone acetylation, another modification of histones, regulates the function of targeted genes via HDAC and histone acetyltransferases (HATs). HDAC inhibitors (HDACi) induce cancer cell cycle arrest, differentiation, and death; reduce angiogenesis; and modulate immune responses [76]. A previous study found that the HDACi panobinostat can downregulate EZH2 and other core proteins of the PRC2 complex [77]. Co-treatment with DZNep and panobinostat caused further depletion of EZH2 compared to treatment with each agent alone, increasing apoptosis in acute myeloid leukemia (AML). Based on these findings, the combination of DZNep and panobinostat is an effective epigenetic treatment approach against AML cells [78].

For histone modification, increasing methylation level and decreasing acetylation level contribute to gene silencing and thereby induce tumor progression [79]. EZH2 gain-of-function mutations as well as CREBBP and EP300 inactivating histone acetyltransferases mutations are implicated in the pathogenesis of germinal center (GC)-derived lymphomas. Recent research showed that co-treatment with EZH2i and HDACi has synergistic effect in GC-derived lymphoma cell lines. Combination of the EZH2i GSK126 and the HDACi romidepsin disrupts the integrity of the PRC2 complex via promotes acetylation of RbAP 46/48 and inhibition of EZH2 enzyme activity, leading to decreased methylation and increased acetylation of H3K27. Combination treatment effectively inhibits proliferation and induces apoptosis in lymphoma cell lines in vitro. Moreover, the combination of GSK126 and romidepsin can not only inhibit tumor growth but also improve overall survival in the SU-DHL-10 xenograft model. This finding is corroborated by MetaVIPER analysis, because synergistic cell lines share a common basal gene expression level and protein activity profile, as confirmed by the TCGA database and cBioPortal [80].

In addition, the synergistic effect has also been reported in the NSCLC model. A study showed that co-treatment with EZH2i DZNep and the HDACi vorinostat synergistically suppressed the proliferation of all tested NSCLC cell lines, regardless of their *EGFR* status [81]. Synergistic effects were observed in a model of small cell carcinoma of the ovary consequently in vivo and in vitro [82]. These synergistic effects were associated with decreased H3K27me3, increased histone acetylation, and depletion of EZH2 and other PRC2 components. Therefore, decreasing EZH2 enzyme activity and its expression are the main goals for suppressing cancer progression.

### EZH2i combined with endocrine therapy

Endocrine therapy, a major modality in the treatment of hormone receptor-positive breast cancer [83] and PCa [84], has achieved good clinical results. However, patients with PCa still progress to CRPC and the cancer becomes untreatable. Enzalutamide is a typical anti-androgen drug for treating PCa that can block androgen binding to the AR as well as prevent AR nuclear translocation and AR-mediated DNA binding. However, a considerable proportion of tumors eventually develop resistance during enzalutamide treatment. A recent study has shown that EZH2 inhibition/depletion enhances the efficacy of enzalutamide in enzalutamide-resistant PCa cells and xenograft models. Moreover, EZH2 and AR are up-regulated in enzalutamide-resistant PCa cells. Mechanically, in enzalutamide-resistant cells, EZH2 remains an epigenetic silencer occupying the AR promoter region and blocking AR target genes, such as *PSA* and *TMPRSS2*. Therefore, EZH2 inhibition activates AR pathways, resulting in a synergistic effect on the inhibition of EZH2 and enzalutamide in resistant cells [85, 86].

Antisense oligonucleotides (ASO), which have been identified as novel drug candidates to ablate EZH2, exert the same effect on EZH2i. Research suggests that combination treatment with ASO targeting EZH2 and AR transcripts has a better effect on inhibiting PCa cell growth, both in vitro and in vivo*,* than monotherapy with these agents [87]. These studies indicate that EZH2 is a critical epigenetic regulator involved in anti-androgen therapy resistance. At the same time, these studies provide a rationale for the combination of enzalutamide with EZH2i. Notably, the study of clinical treatment with the EZH2i tazemetostat and enzalutamide in patients with CRPC has entered clinical trials recently (NCT04179864).

The estrogen receptor (ER) is the most important factor in the pathophysiology of breast cancer [88]. Therefore, modulation of ER activity has been exploited to develop drugs against ER^+^ breast cancer, such as tamoxifen, representing important endocrine therapies [89].

Epigenetic reprogramming also influences ERs. A recent study discovered that tamoxifen resistance in breast cancer is driven by the epigenetic regulator EZH2, which silences the expression of the growth regulation by estrogen in breast cancer 1(*GREB1*), an ERα co-factor that is a critical regulatory protein of the ERα pathway. The GREB1-ERα axis plays a pivotal role in determining cell fate in response to anti-estrogen treatment. Researchers have observed a dramatic increase in EZH2 protein levels in clinical samples that are resistant to tamoxifen compared to clinical samples that are sensitive, suggesting that EZH2 causes tamoxifen resistance in breast cancer. Pharmacologic inhibition of EZH2 by GSK126 and tazemetostat retard the growth of xenograft tumors in mouse models, although GREB1 expression continues to increase. The negative correlation of EZH2 with GREB1 can also predict patient responses to endocrine therapy. Interestingly, EZH2-negative and GREB1-positive patients have better clinical outcomes compared to EZH2-positive and GREB1-negative tumors, and the combination of EZH2i with tamoxifen has synergistic effects [90].

## Conclusions

Epigenetic regulation usually has a profound impact on the whole transcriptome and causes further global alteration in upstream transcription. Alterations induced by EZH2i may have favorable as well as unfavorable consequences in cancer treatment. For instance, EZH2i improves the infiltration of suppressive immune cells in the tumor microenvironment but induces chemotherapy and endocrine therapy resistance. Therefore, combination with other therapies may be an effective way to exert synergistic and compensatory effects in different cancer types to improve the final clinical outcome. Our previous study also summarized the correlation between EZH2 and the key genes involved cellular metabolic pathways to propose a potential strategy that combination of EZH2i with metabolic regulators will be useful for future cancer therapy [91]. One of the more canonical study demonstrated that a triple combination therapy with EZH2, BRD4, and MAPK pathway inhibitors showed significantly improve the treatment efficacy of solid tumor models with abnormally high levels of EZH2 [92].

To determine whether EZH2i should be used for priming, intermittently with other methods, or sequentially in combination therapies, the constituent treatment strategies need to be precisely and comprehensively studied. A better understanding of the therapeutic mechanisms, either alone or in combination, can further facilitate the development of a strategy to utilize EZH2i easily, effectively and safely with other medications or therapies.

## Data Availability

Not applicable.

## Abbreviations

EZH2: Enhancer of zeste homolog 2
EZH2i: Enhancer of zeste homolog 2 inhibitors
FDA: Food and Drug Administration
MTD: Maximum-tolerated dose
PRC2: Polycomb repressive complex 2
Tyr641: Tyrosine 641 site
FL: Follicular lymphoma
GCB: Germinal center B-cell
DLBCL: Diffuse large B-cell lymphoma
NHL: Non-Hodgkin’s lymphoma
SAM: *S*-AdenosylMethionine
SAH: *S*-Adenosylhomocysteine
MM: Multiple myeloma
CRPC: Castration resistant prostate cancer
mCRPC: Metastatic castration resistant prostate cancer
AR: Androgen receptor
PD-L1: Programmed cell death ligand-1
TGF-β: Transforming growth factor-β
IGF-1R: Insulin-like growth factor 1 receptor
PI3K: Phosphoinositide-3-kinase
DNMT1: DNA methyltransferase 1
ICB: Immune checkpoint blockade
Anti-PD-1: Anti-programmed cell death-1
Anti-PD-L1: Anti-programmed cell death-ligand 1
Anti-CTLA-4: Anti-cytotoxic T lymphocyte antigen 4
IFN-γ: Interferon-γ
TNF-α: Tumor necrosis factor-α
IRF1: Interferon regulatory factor 1
HNSCC: Head and neck squamous cell carcinoma
MDSCs: Myeloid-derived suppressor cells
PCa: Prostate cancer
NSCLC: Non-small cell lung cancers
EGFR: Epidermal growth factor receptor
Doc: Docetaxel
TMZ: Temozolomide
HCC: Hepatocellular carcinoma
ATM: Ataxia telangiectasia mutated
FOXO3: Forkhead box O3
PAR: Poly ADP-ribose
PARP: Poly ADP-ribose polymerase
PARPi: Poly ADP-ribose polymerase inhibitors
PARylation: Poly ADP-ribosylation
HR: Homologous recombination
HDAC: Histone deacetylases
HDACi: Histone deacetylases inhibitors
HATs: Histone acetyltransferases
AML: Acute myeloid leukemia
GC: Germinal center
ASO: Antisense oligonucleotides
ER: Estrogen receptor
GREB1: Growth regulation by estrogen in breast cancer 1
ER: Estrogen receptor
